# Surfer’s myelopathy in Bali: a case series of three patients and review of the pathophysiology, proposed diagnostic criteria, and treatment

**DOI:** 10.1186/s41016-026-00450-w

**Published:** 2026-09-15

**Authors:** Mira Yuniarti, Lorentia Harisusanti, Jeffrey Tanudjaja, Gilbert Sterling Octavius

**Affiliations:** Pelita Harapan University, Tangerang, Indonesia

**Keywords:** Surfer’s myelopathy, Nontraumatic spinal cord injury, Acute myelopathy, Spinal cord ischemia, Magnetic resonance imaging, Indonesia

## Abstract

**Background:**

Surfer’s myelopathy is a rare, nontraumatic spinal cord injury that predominantly affects young, novice surfers after prolonged spinal hyperextension. Owing to its low incidence and heterogeneous clinical and imaging features, the condition remains underrecognized, and its pathophysiology, diagnostic criteria, and optimal management strategies are not fully established. Increased awareness is particularly important in popular surfing destinations.

**Case presentation:**

We report three cases of surfer’s myelopathy in previously healthy young women who developed acute lower back pain followed by rapidly progressive bilateral lower-limb weakness and urinary retention shortly after recreational surfing. None reported trauma or preceding illness. Neurological examination revealed paraparesis with preserved upper-limb function, corresponding to American Spinal Cord Injury Association Impairment Scale grade D in all patients. Thoracic magnetic resonance imaging performed within 10–18 h after symptom onset demonstrated central intramedullary T2-weighted hyperintensity extending from the mid-thoracic spinal cord toward the conus medullaris, without evidence of compressive pathology. Management consisted of hemodynamic optimization, intravenous corticosteroids, spinal immobilization, and supportive care. All patients showed partial neurological improvement before discharge.

**Conclusion:**

Surfer’s myelopathy should be considered in young patients presenting with acute nontraumatic paraparesis and autonomic dysfunction following surfing. Characteristic MRI findings support the diagnosis and help exclude alternative etiologies. Our cases support the applicability of broader diagnostic criteria and highlight the importance of early recognition, supportive management, and preventive education, particularly in regions with high surfing activity.

## Background

Spinal cord injury occurring in the absence of structural spinal trauma is an uncommon cause of neurological impairment during recreational activities. This form of nontraumatic spinal cord injury related to surfing has been previously described as surfer’s myelopathy [1]. First-time surfers, defined as those with no prior experience on a surfboard before the day of injury, are at particularly high risk and account for approximately 89.5–100% of novice surfers reported across published studies [2]. The incidence of surfer’s myelopathy has been estimated at 2.2 to 6.6 injuries per 1000 h of surfing [3], and predominantly affects younger individuals, with a mean age of 25.9 years and an age range spanning from 15 to 46 years [4]. However, accumulating evidence suggests that this condition can also occur in children and adolescents and is not intrinsically related to surfing itself, but rather to prolonged spinal hyperextension and associated vascular compromise, despite its name [5].

Fewer than 100 cases have been reported since the first description in 2004, and current evidence consists predominantly of case reports and small case series [6, 7]. This case series presents three cases of surfer’s myelopathy in Bali and includes a literature review to summarize the current state of knowledge in this field.

### Case presentation

**Fig. 1:**
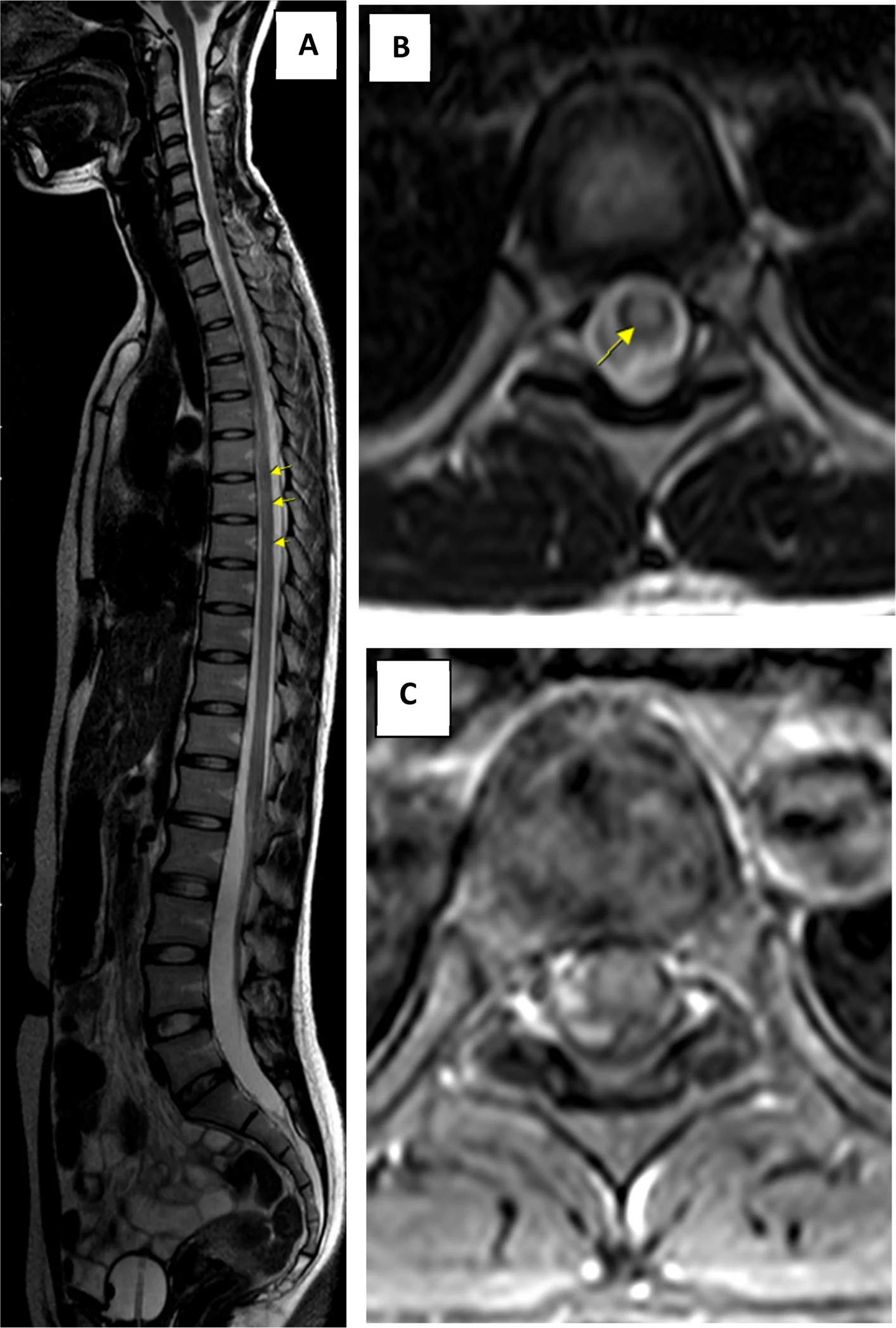
**A** Whole-spine sagittal T2-weighted imaging (T2WI) demonstrates a faint hyperintense lesion at the T6–T7 and T7–T8 levels (arrows), which is more clearly visualized on the corresponding axial image (**B**, arrow). No contrast enhancement is observed on post-contrast imaging (**C**)

The first case is a 24-year-old Korean woman who presented approximately 15 h after the onset of progressive bilateral lower-limb weakness that began the previous evening. The patient had surfed for approximately 3 h earlier that morning. After approximately 3 h of surfing, she developed acute lower back pain followed by progressive bilateral lower-limb weakness and urinary retention. Neurological examination demonstrated reduced motor strength in the lower extremities, with a Medical Research Council (MRC) grade of 4/5 in the right lower limb and 3/5 in the left lower limb, while upper-extremity strength remained normal. Sensory examination demonstrated hypoesthesia below T9. Bladder dysfunction was present in the form of acute urinary retention requiring catheterization. According to the American Spinal Cord Injury Association (ASIA) Impairment Scale (AIS), the patient was classified as AIS grade D on admission. Laboratory investigations, including cerebrospinal fluid (CSF) analysis, were within normal limits. Contrast-enhanced thoracic magnetic resonance imaging (MRI) 18 h after the first onset, with multiplanar T1-weighted, T2-weighted, and short tau inversion recovery (STIR) sequences, revealed a mildly hyperintense intramedullary lesion at the T6–T7 and T7–T8 levels, consistent with short-segment myelopathy (Fig. 1A–C). The patient received intravenous corticosteroids and supportive care with partial neurological improvement before discharge after 7 days (AIS D).

**Fig. 2:**
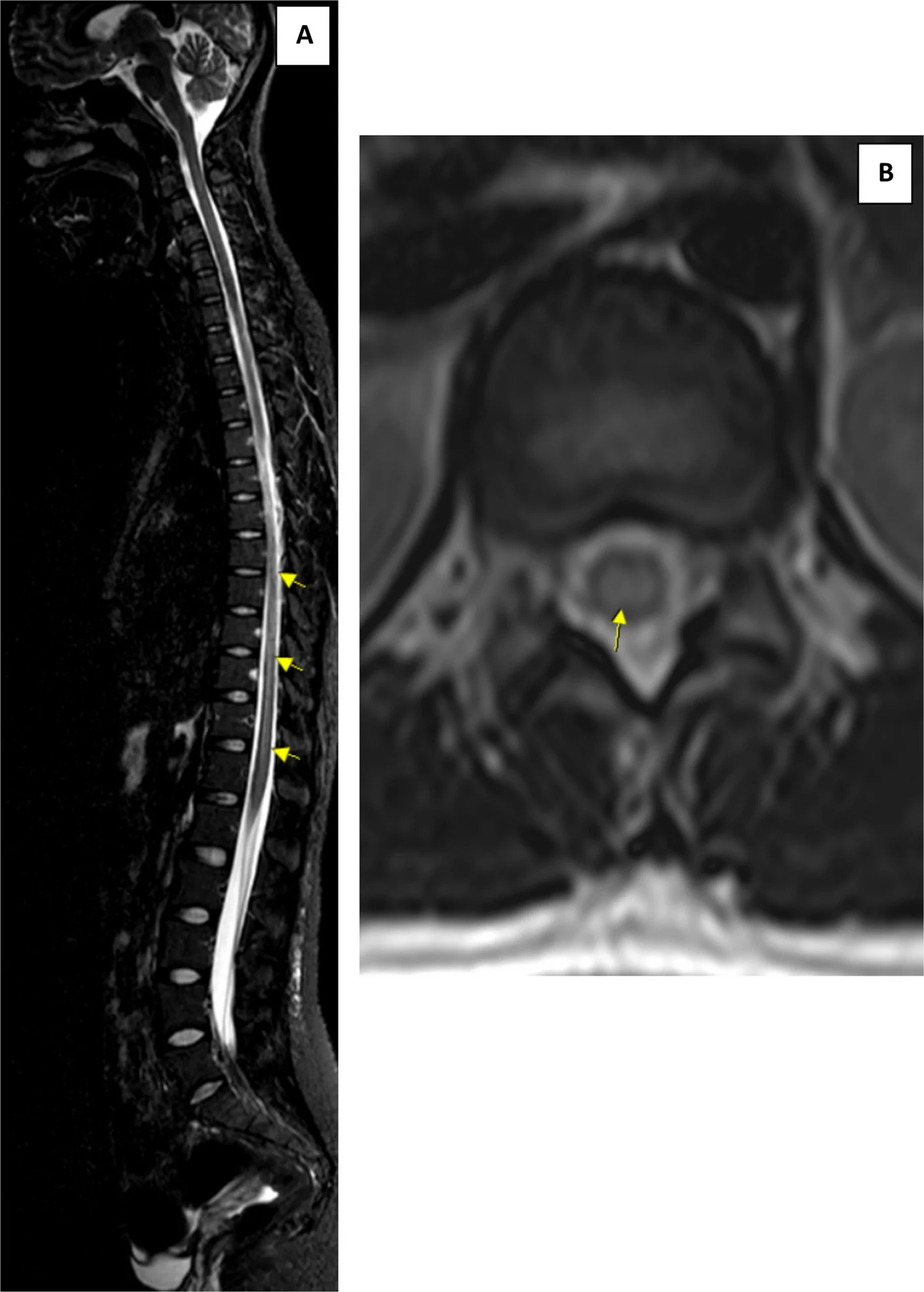
**A** Sagittal whole-spine short-tau inversion recovery (STIR) and **B** axial T2WI images reveal an extensive central long-segment hyperintense lesion intramedullary extending from T8 to T12

The second case is a 20-year-old previously healthy Chinese woman who presented to the emergency department with acute bilateral lower-limb weakness and urinary retention. She developed acute lower back pain while surfing, followed 6 h later by progressive bilateral lower-limb weakness and urinary retention. Neurological assessment demonstrated bilateral lower-extremity motor weakness (MRC grade 4 bilaterally), while upper-extremity motor and sensory functions were preserved. Bladder dysfunction was present, requiring urinary catheterization due to acute urinary retention. The patient was classified as AIS grade D on admission. Her non-contrast thoracolumbar MRI performed 10 h after onset, with our usual institutional protocol, reveals a central T2 hyperintense lesion extending from T8 to T12, suggestive of a long-segment myelopathy (Fig. 2A, B). She received supportive treatment including corticosteroids, hemodynamic optimization, and spinal immobilization, with partial neurological improvement before discharge.

**Fig. 3:**
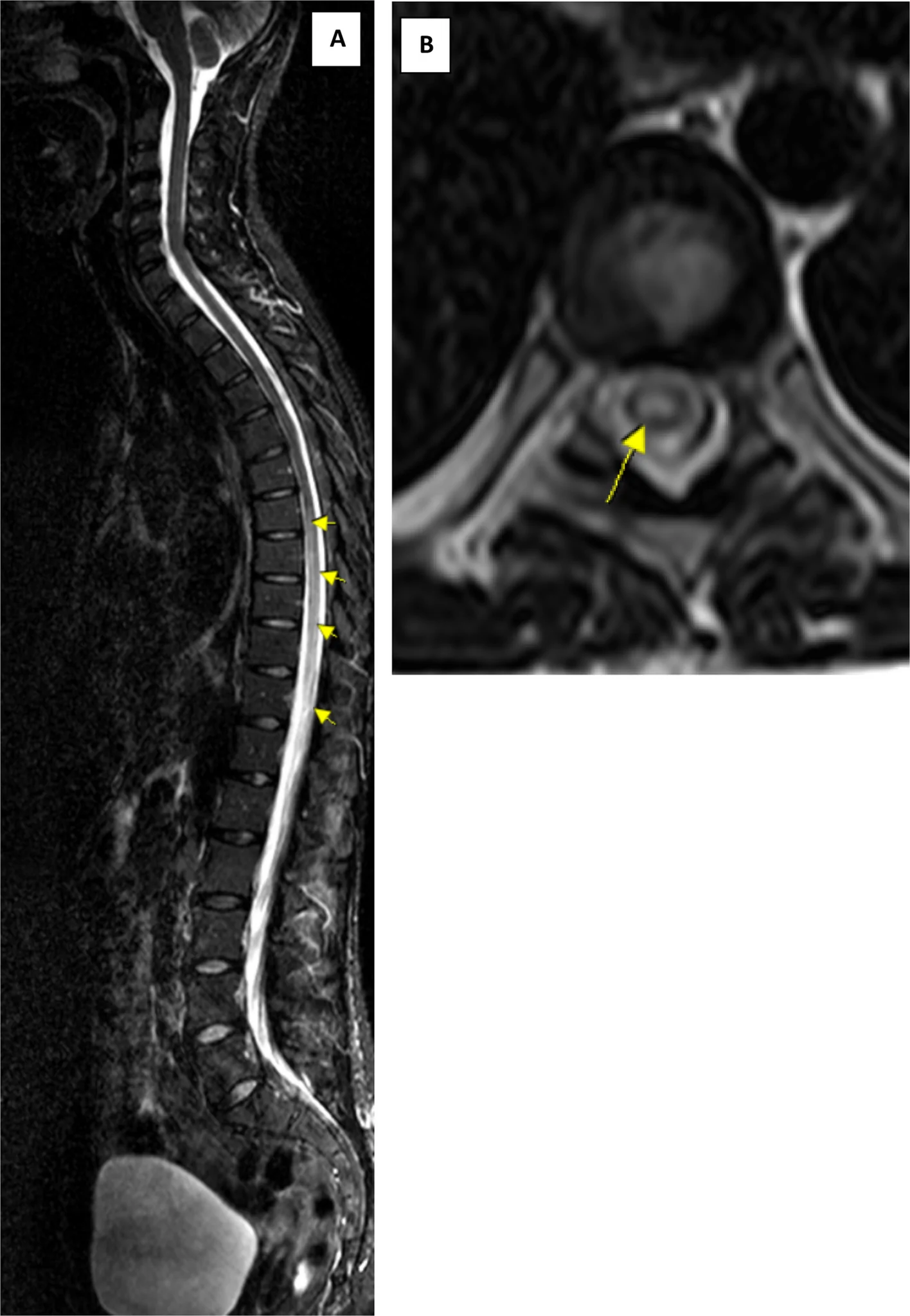
**A** Sagittal whole-spine short-tau inversion recovery (STIR) and axial **B** T2WI images reveal an extensive central long-segment hyperintense lesion intramedullary extending from T7 to T11

The last case is a 25-year-old previously healthy Caucasian woman who presented to the emergency department 7 h after the onset of acute lower back pain followed by rapidly progressive bilateral lower-limb weakness and urinary retention. Following her first surfing lesson, she developed acute lower back pain followed by rapidly progressive bilateral lower-limb weakness and urinary retention. Neurological assessment revealed bilateral lower-extremity motor weakness (MRC grade 4 bilaterally), while upper-extremity motor was preserved. Sensory examination demonstrated reduced sensation below approximately the T9 dermatome. Bladder dysfunction was present, manifested as acute urinary retention requiring catheterization. The patient was classified as AIS grade D on admission. Non-contrast thoracolumbar spine MRI with the usual protocol was performed 12–13 h after onset and demonstrated a central intramedullary T2 hyperintense lesion extending from the T7–T8 to T10–T11 levels, without evidence of compressive pathology, findings consistent with surfer’s myelopathy (Fig. 3A and B). She received supportive treatment including corticosteroids, hydration, blood pressure optimization, and spinal immobilization, with partial neurological improvement before discharge.

## Discussion

This case series describes three cases of surfer’s myelopathy in women who were recreational surfers. The non-traumatic, sudden onset of paraparesis in otherwise healthy individuals strongly supports this diagnosis. In our series, neurological symptoms developed within hours after surfing, and an MRI was performed within 10–18 h of symptom onset, which is consistent with the temporal pattern described in previous reports of surfer’s myelopathy.

### Pathophysiology

Surfer’s myelopathy is hypothesized to result from sustained hyperextension of the spine, leading to vascular compromise and subsequent ischemia of the distal spinal cord [1, 3]. The clinical presentation, together with characteristic imaging findings, supports a vascular etiology involving dynamic arterial compression, vasospasm, or thrombotic infarction of the great anterior radicular artery of Adamkiewicz [1, 3]. This artery typically originates from an inferior intercostal or lumbar artery between T9 and L2, ascends to anastomose with the anterior spinal artery, and supplies the inferior portion of the spinal cord. The suggested risk factors include a slender body build with relatively underdeveloped musculature, dehydration, and a hypercoagulable state associated with prolonged travel [1, 3].

Infarction of this vessel, as observed in conditions such as aortic aneurysm injury or repair, produces a similar vascular distribution of spinal cord injury on MRI to that seen in surfer’s myelopathy [3]. The artery of Adamkiewicz supplies the lower thoracic cord and conus medullaris, corresponding to the typical MRI distribution [1, 3].

Alternative mechanisms, including fibrocartilaginous embolism [3], arterial dissection [8], venous infarction [1], and mechanical cord compression [9] have been proposed but remain unproven. Overall, the ischemic hypothesis remains the most widely accepted.

### Radiology findings

Spinal MRI findings in surfer’s myelopathy typically show longitudinally extensive T2-weighted hyperintensity extending from the mid-thoracic spine (approximately T5–T10) to the conus medullaris within 2–24 h of symptom onset, often accompanied by variable fusiform cord enlargement, in a pencil-like fashion [10, 11]. The differing extents of thoracic spinal cord involvement may be attributed to anatomical variability in the origin of the artery of Adamkiewicz, which arises from T5 to T8 in approximately 15% of individuals, from T9 to T12 in about 75%, and from L1 to L2 in the remaining 10% of the population [11]. MRI typically demonstrates longitudinally extensive central T2 hyperintensity extending from the mid-thoracic cord to the conus within 24 h [11]. The role of DWI remains uncertain. Although diffusion restriction may indicate acute spinal cord ischemia, inconsistent findings and technical limitations prevent its routine use in surfer’s myelopathy [12, 13].

### Diagnosis

The diagnosis of surfer’s myelopathy hinges on the rapid onset of signs and symptoms after a known insult, with all of the other plausible mechanisms being excluded. The most frequently reported manifestations of surfer’s myelopathy include back pain, urinary dysfunction such as incontinence or retention, paraplegia or paraparesis, lower extremity weakness, and sensory deficits. Because clinical presentations vary across cases, additional neurological findings may include hypesthesia, hypoalgesia, and hyperesthesia [3].

Two authors [4, 5] attempt to suggest diagnostic criteria for surfer’s myelopathy (Table 1). All three patients fulfilled the recently proposed Alva-Díaz diagnostic criteria. The diagnosis was based on the characteristic clinical presentation, MRI findings, and exclusion of compressive, infectious, and inflammatory causes [2, 3, 14].

**Table 1:** Two proposed diagnostic criteria for surfer’s myelopathy

Authors	Criteria
Albuja et al. (2017) [5]	1. Younger than 40 years 2. Acute onset of paraparesis within 24 h following a hyperextension injury 3. Initial spinal MRI showing diffusion restriction, characterized by hyperintensity on diffusion-weighted imaging with corresponding hypointensity on the apparent diffusion coefficient map 4. Follow-up MRI revealed hyperintensity on T2-weighted sequences
Alva-Diaz et al. (2022) [4]	1. A precipitating activity resulting in nontraumatic hyperextension of the back or spine 2. Hyperacute onset, defined as the development of motor or sensory deficits within 4 h or less following the hyperextension event 3. Clinical signs and symptoms of spinal cord injury, including acute pain that may precede neurological deficits, as well as motor, sensory, or autonomic dysfunction corresponding to the affected spinal level 4. Magnetic resonance imaging demonstrating central spinal cord involvement, characterized by diffuse hyperintense T2-weighted signal within the central cord that may extend to the conus medullaris, with an infarction-like pattern or longitudinally extensive “pencil-like” or “owl-eye/snake-eye” T2 hyperintensities 5. Absence of an alternative diagnosis that could explain the clinical and imaging findings Definite case: meets all criteria Probable case: At least 1, 2, and 3 Possible case: At least 1 and 3

### Differential diagnosis

The principal differential diagnosis for MRI findings demonstrating signal abnormalities confined to the gray matter is acute spinal cord infarction. Other important considerations include transverse myelitis associated with neuromyelitis optica (NMO) or autoimmune disorders such as systemic lupus erythematosus (SLE). Although these conditions often present with preceding inflammatory signs and symptoms and show differing imaging characteristics, their initial presentations may be severe and radiologically similar to surfer’s myelopathy [10]. Important differentials include transverse myelitis, neuromyelitis optica, and multiple sclerosis [11]. Acute spinal cord infarction remains the closest radiological differential [15].

### Treatment

Management is largely supportive and includes maintenance of spinal cord perfusion, rehabilitation, and individualized corticosteroid use [16]. Besides supportive therapy, intense rehabilitation and physical therapy in a qualified spinal cord injury center have been the hallmark of clinical therapy [13]. Intravenous corticosteroids were commonly used as the main treatment, despite the lack of supporting evidence. Their administration was associated with an improvement of at least one AIS grade in 55% of reported cases. However, the American Academy of Neurological Surgeons advises against the use of methylprednisolone in acute spinal cord injury, even though several case reports have described its use in treating acute hyperextension-induced myelopathy [4]. Assuming the pathophysiology is of an ischemic nature, steroids will not be effective in surfer’s myelopathy [1, 17].

A better understanding of surfer’s myelopathy, together with heightened clinical awareness, may facilitate earlier recognition and intervention [6]. Prevention in the form of public service announcements or increasing awareness among physicians, novice athletes, and surfing schools has therefore been advocated as the most effective approach to managing this condition [2]. Karabegovic et al. proposed the Surfer’s Myelopathy Foundation guidelines for safe surfing, summarized by the mnemonic “SPINE”: Sit on the board while waiting for waves, Pace time in the water with a recommended limit of approximately 30 min, Insist on instruction from a knowledgeable surfing instructor, Notice early signs of back pain or discomfort, and Exit the water to seek medical attention if pain, tingling, or weakness develops [18].

### Prognosis

The prognosis of surfer’s myelopathy remains incompletely defined and appears to vary widely. Case reports suggest that patients presenting with milder symptoms and less severe weakness may demonstrate early neurological improvement during hospitalization or rehabilitation. However, many patients still require assistive devices for mobility at discharge, and the extent of long-term functional recovery remains variable [6]. Patients presenting with complete paraplegia generally have poorer recovery, whereas milder deficits are associated with better functional outcomes after rehabilitation [10].

### Limitation

One limitation of this case series is the absence of long-term clinical and radiological follow-up. All three patients were international travelers who returned to their home countries shortly after hospital discharge, and subsequent follow-up evaluations could not be obtained despite attempts through available contact channels. Consequently, the long-term neurological outcome and potential radiological evolution of the spinal cord lesions could not be assessed. All three patients returned to their home countries after discharge, preventing long-term clinical and MRI follow-up.

## Conclusion

Surfer’s myelopathy is a rare nontraumatic spinal cord injury that typically affects young novice surfers after prolonged spinal hyperextension. Our three cases highlight the characteristic clinical presentation of acute paraparesis with autonomic dysfunction and the typical MRI finding of central thoracic intramedullary T2 hyperintensity without compressive pathology. Early recognition and prompt supportive management are essential, although no standardized treatment has yet been established. Increased awareness among clinicians, radiologists, and surfing instructors, particularly in popular surfing destinations such as Bali, may facilitate earlier diagnosis and promote preventive strategies.

## Data Availability

Available upon request.

## Abbreviations

AANS: American Association of Neurological Surgeons
ADC: Apparent diffusion coefficient
AIS: American Spinal Cord Injury Association Impairment Scale
AQP: Aquaporin
CCS: Central cord syndrome
CNS: Congress of Neurological Surgeons
CSF: Cerebrospinal fluid
CT: Computed tomography
DWI: Diffusion-weighted imaging
FCE: Fibrocartilaginous embolism
LETM: Longitudinally extensive transverse myelitis
MAP: Mean arterial pressure
MRI: Magnetic resonance imaging
MRC: Medical research council
NMO: Neuromyelitis optica
SCIWORA: Spinal cord injury without radiographic abnormality
SLE: Systemic lupus erythematosus
STIR: Short tau inversion recovery
T1WI: T1-weighted imaging
T2WI: T2-weighted imaging
tPA: Tissue plasminogen activator
